# Orthogonal outlier detection and dimension estimation for improved MDS embedding of biological datasets

**DOI:** 10.3389/fbinf.2023.1211819

**Published:** 2023-08-10

**Authors:** Wanxin Li, Jules Mirone, Ashok Prasad, Nina Miolane, Carine Legrand, Khanh Dao Duc

**Affiliations:** ^1^ Department of Computer Science, University of British Columbia, Vancouver, BC, Canada; ^2^ Department of Mathematics, University of British Columbia, Vancouver, BC, Canada; ^3^ Centre de Mathématiques Appliquées, Ecole Polytechnique, Palaiseau, France; ^4^ Department of Chemical and Biological Engineering, School of Biomedical Engineering, Colorado State University, Fort Collins, CO, United States; ^5^ Department of Electrical and Computer Engineering, University of California, Santa Barbara, Santa Barbara, CA, United States; ^6^ Université Paris Cité, Génomes, biologie cellulaire et thérapeutique U944, INSERM, CNRS, Paris, France

**Keywords:** orthogonal outliers, outlier detection, outlier correction, multidimensional scaling, shape data, microbiome data, scRNA seq

## Abstract

Conventional dimensionality reduction methods like Multidimensional Scaling (MDS) are sensitive to the presence of orthogonal outliers, leading to significant defects in the embedding. We introduce a robust MDS method, called *DeCOr-MDS* (Detection and Correction of Orthogonal outliers using MDS), based on the geometry and statistics of simplices formed by data points, that allows to detect orthogonal outliers and subsequently reduce dimensionality. We validate our methods using synthetic datasets, and further show how it can be applied to a variety of large real biological datasets, including cancer image cell data, human microbiome project data and single cell RNA sequencing data, to address the task of data cleaning and visualization.

## 1 Introduction

Multidimensional scaling (MDS) is a commonly used and fast method of data exploration and dimension reduction, with the unique capacity to take non-euclidean dissimilarities as its input. However, sensitivity to outliers is a major drawback (Harmeling et al., 2005; Blouvshtein and Cohen-Or, 2019). As arbitrary removal of outliers is undesirable, a possible alternative is to detect outliers and accommodate their influence on the MDS embedding, thus leveraging the information contained in outlying points.

Outlier detection has been widely used in biological data. Sheih and Yeung proposed a method using principal component analysis (PCA) and robust estimation of Mahalanobis distances to detect outlier samples in microarray data (Shieh and Hung, 2009). Chen *et al.* reported the use of two PCA methods to uncover outlier samples in multiple simulated and real RNA-seq data (Oh et al., 2008). Outlier influence can be mitigated depending on the specific type of outlier. In-plane outliers and bad leverage points can be harnessed using *ℓ*_1_-norm (Spence and Lewandowsky, 1989; Cayton and Dasgupta, 2006; Forero and Giannakis, 2012), correntropy or M-estimators (Mandanas and Kotropoulos, 2017). Outliers which violate the triangular inequality can be detected and corrected based on their pairwise distances (Blouvshtein and Cohen-Or, 2019). Orthogonal outliers are another particular case, where outliers have an important component, orthogonal to the hyperspace where most data is located. These outliers often do not violate the triangular inequality, and thus require an alternative approach.

Although MDS is known to be sensitive to such orthogonal outliers (Song et al., 2007; Legrand, 2017), none of the existing methods addresses this issue, to the best of our knowledge. We present here a robust MDS method, called *DeCOr-MDS*, Detection and Correction of Orthogonal outliers using MDS. *DeCOr-MDS* takes advantage of geometrical characteristics of the data to reduce the influence of orthogonal outliers, and estimate the dimension of the dataset. Our paper is organized as follows. We first describe the procedure and its implementation in detail. We then validate our method on synthetic data to confirm the accuracy and characterize the importance of different parts of our procedure. We further run the method on different experimental datasets from single cell images, microbiome sequencing data, and scRNA-seq data. Our experiments show that *DeCOr-MDS* can detect artefacts in cell shape data, improve the visualization of clusters in microbiome data, and be used as a step for quality control for scRNA-seq data, illustrating how it can be broadly applied to interpret and improve the performance of MDS on biological datasets. Finally, we discuss the advantages and limitations of our method and future directions.

## 2 Materials and methods

### 2.1 Background: height and volume of n-simplices

We recall some geometric properties of simplices, which our method is based on. For a set of *n* points (*x*_1_, … , *x*_*n*_), the associated *n*-simplex is the polytope of vertices (*x*_1_, … , *x*_*n*_) (a 3-simplex is a triangle, a 4-simplex is a tetrahedron and so on). The height *h*(*V*_*n*_, *x*) of a point *x* belonging to a *n*-simplex *V*_*n*_ can be obtained as (Sommerville, 1929)

$$h\left(V_{n},x\right)=n\frac{V_{n}}{V_{n-1}},$$ (1)

where *V*_*n*_ is the volume of the *n*-simplex, and *V*_*n*−1_ is the volume of the (*n* − 1)-simplex obtained by removing the point *x*. *V*_*n*_ and *V*_*n*−1_ can be computed using the pairwise distances only, with the Cayley-Menger formula (Sommerville, 1929):

$$V_{n}=\sqrt{\frac{\left|\det\left(CM_{n}\right)\right|}{2^{n}\cdot {\left(n!\right)}^{2}}},$$ (2)

where *det*(*CM*_*n*_) is the determinant of the Cayley-Menger matrix *CM*_*n*_, that contains the pairwise distances $d_{i,j}=\left\|x_{i}-x_{j}\right\|$ , as

$$CM_{n}=\left[\begin{array}{cccccc} 0 & 1 & 1 & \ldots & 1 & 1\\ 1 & 0 & d_{1,2}^{2} & \ldots & d_{1,n}^{2} & d_{1,n+1}^{2}\\ 1 & d_{2,1}^{2} & 0 & \ldots & d_{2,n}^{2} & d_{2,n+1}^{2}\\ \ldots & \ldots & \ldots & \ldots & \ldots & \ldots \\ 1 & d_{n,1}^{2} & d_{n,2}^{2} & \ldots & 0 & d_{n,n+1}^{2}\\ 1 & d_{n+1,1}^{2} & d_{n+1,2}^{2} & \ldots & d_{n+1,n}^{2} & 0 \end{array}\right].$$ (3)

### 2.2 Orthogonal outlier detection and dimensionality estimation

**FIGURE 1:**
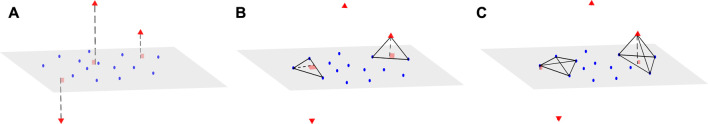
Example of a dataset with orthogonal outliers and n-simplices. **(A)** Representation of a dataset with regular data points (blue) belonging to a main subspace of dimension 2 with some noise, and orthogonal outliers (red triangle symbols) in the third dimension. **(B)** View of two instances of 3-simplices (triangles), one with only regular points (left) and the other one containing one outlier (right). The height drawn from the outlier is close to the height of the regular triangle. **(C)** Upon adding other regular points to obtain tetrahedrons (4-simplices), the height drawn from the outlier (right) becomes significantly larger than the height drawn from the same point (left) as in **(B)**.

We now consider a dataset **X** of size *N* × *d*, where *N* is the sample size and *d* the dimension of the data. We associate with **X** a matrix **D** of size *N* × *N*, which represents all the pairwise distances between observations of **X**. We also assume that the data points can be mapped into a vector space with *regular observations* that form a *main* subspace of unknown dimension d* with some small noise, and additional *orthogonal outliers* of relatively large orthogonal distance to the main subspace (Figure 1A). Our proposed method aims to infer from **D** the dimension of the main data subspace d*, using the geometric properties of simplices with respect to their number of vertices: Consider a (*n* + 2)-simplex containing a data point *x*_*i*_ and its associated height, that can be computed using Eq. 1 in Section 2.1. When *n* < d* and for *S* large enough, the distribution of heights obtained from different simplices containing *x*_*i*_ remains similar, whether *x*_*i*_ is an orthogonal outlier or a regular observation (see Figure 1B). In contrast, when *n* ≥ d*, the median of these heights approximately yields the distance of *x*_*i*_ to the main subspace (Figure 1C). This distance should be significantly larger when *x*_*i*_ is an orthogonal outlier, compared with regular points, for which these distances are tantamount to the noise.

To estimate d* and for a given dimension *n* tested, we thus randomly sample, for every *x*_*i*_ in **X**, *S*(*n* + 2)-simplices containing *x*_*i*_, and compute the median of the heights $h_{i}^{n}$ associated with these *S* simplices. Upon considering, as a function of the dimension *n* tested, the distribution of median heights $\left(h_{1}^{n},\ldots ,h_{N}^{n}\right)$ (with 1 ≤ *i* ≤ *N*), we then identify d* as the dimension at which this function presents a sharp transition towards a highly peaked distribution at zero. To do so, we compute ${\tilde{h}}_{n}$ , as the mean of $\left(h_{1}^{n},\ldots ,h_{N}^{n}\right)$ , and estimate d* as

$$\bar{n}=\underset{n}{\mathrm{argmax}}\frac{{\tilde{h}}_{n-1}}{{\tilde{h}}_{n}}.$$ (4)

Furthermore, we detect orthogonal outliers using the distribution obtained in $\bar{n}$ , as the points for which $h_{i}^{\bar{n}}$ largely stands out from ${\tilde{h}}_{\bar{n}}$ . To do so, we compute $\sigma_{\bar{n}}$ the standard deviation observed for the distribution $\left(h_{1}^{\bar{n}},\ldots ,h_{N}^{\bar{n}}\right)$ , and obtain the set of orthogonal outliers **O** as

$$O=\left\{i\;|\;h_{i}^{\bar{n}}>{\tilde{h}}_{\bar{n}}+c\times \sigma_{\bar{n}}\right\},$$ (5)

where *c* > 0 is a parameter set to achieve a reasonable trade-off between outlier detection and false detection of noisy observations. Our implementation uses *c* = 3 by default (following the three *σ* rule Pukelsheim (1994), and which corresponds to ∼99.9% of a Gaussian distribution being conserved), value which was also used in our experiments. In case users possess prior information or want to control the fraction of detected outliers, the value of *c* may be modified, with increasing *c* making the detection stricter. Also note that our method introduces another parameter *S*, as it samples *S* simplices to calculate the median of the corresponding heights. Therefore, *S* should be large enough so the resulting sample median well approximates the global median. Assuming the heights being sampled from a continuous distribution, this can be guaranteed as the sample median is asymptotically normal, with mean equal to the true median and the standard deviation proportional to $\frac{1}{\sqrt{S}}$ (Rider, 1960).

### 2.3 Correcting the dimensionality estimation for a large outlier fraction

The method presented in the previous section assumes that at dimension d*, the median height calculated for each point reflects the distance to the main subspace. This assumption is valid when the fraction of orthogonal outliers is small enough, so that the sampled *n*-simplex likely contains regular observations only, aside from the evaluated point. However, if the number of outliers gets large enough so that a significant fraction of *n*-simplices also contains outliers, then the calculated heights would yield the distance between *x*_*i*_ and an outlier-containing hyperplane, whose dimension is larger than a hyperplane containing only regular observations. The apparent dimensionality of the main subspace would thus increase and generates a positive bias on the estimate of d*.

Specifically, if **X** contains a fraction of *p* outliers, and if we consider *o*_*n*,*p*,*N*_ the number of outliers drawn after uniformly sampling *n* + 1 points (to test the dimension *n*), then *o*_*n*,*p*,*N*_ follows a hypergeometric law, with parameters *n* + 1, the fraction of outliers *p* = *N*_*o*_/*N*, and *N*. Thus, the expected number of outliers drawn from a sampled simplex is (*n* + 1) × *p*. After estimating $\bar{n}$ (from Section 2.2), and finding a proportion of outliers $\bar{p}=|O|/N$ using Eq. 5, we hence correct $\bar{n}$ *a posteriori* by substracting the estimated bias *δ*, as the integer part of the expectation of *o*_*n*,*p*,*N*_, so the debiased dimensionality estimate n* is

$$n^{*}=\bar{n}-\left\lfloor \left(\bar{n}+1\right)\times p\right\rfloor .$$ (6)

### 2.4 Outlier distance correction

Upon identifying the main subspace containing regular points, our procedure finally corrects the pairwise distances that contain outliers in the matrix **D**, in order to apply a MDS that projects the outliers in the main subspace. In the case where the original coordinates cannot be used (e.g., as a result of some transformation or if the distance is non Euclidean), we perform the two following steps: 1) We first apply a MDS on **D** to place the points in a euclidean space of dimension *d*, as a new matrix of coordinates $\tilde{X}$ . 2) We run a PCA on the full coordinates of the estimated set of regular data points (i.e., $\tilde{X}\backslash O$ ), and project the outliers along the first ${\bar{n}}^{*}$ principal components of the PCA, since these components are sufficient to generate the main subspace. Using the projected outliers, we accordingly update the pairwise distances in **D** to obtain the corrected distance matrix D*. Note that in the case where **D** derives from a euclidean distance between the original coordinates, we can skip step 1), and directly run step 2) on the full coordinates of the estimated set of regular data points.

### 2.5 Overall procedure and implementation

The overall procedure, called DeCOr-MDS, is described in Algorithm 1. The values for the parameters *S* and *c* were set by default and in our experiments to *S* = 100 and *c* = 3. We also provide an implementation in Python 3.8.10 available on this github repository: https://github.com/wxli0/DeCOr-MDS.

Algorithm 1 DeCOr-MDS.

**Input** *D* the pairwise distance matrix of the dataset of size *N* × *d*, *E*_dim_ the set of dimensions (≤d) to be tested, *c* and *S* user-specified constants
**Output** ${\bar{n}}^{*}$ the relevant dimension of the dataset, *O* the list of orthogonal outliers, and D* the matrix of corrected pairwise distances
**for** *n* in *E*_*dim*_ **do**
 **for** i in [1,N] **do**
  **for** j in [1,S] **do**
   Sample a (*n* + 2)-simplex *V*_*i*,*j*_ containing *x*_*i*_
   Compute the height (using *D* and Eq. 1) $h_{i}^{\left(j,n\right)}≔h_{i}^{j}\left(V_{i,j},x_{i}\right)$

  **end for**

$h_{i}^{n}≔median\left(h_{i}^{\left(1,n\right)},h_{i}^{\left(2,n\right)},\ldots ,h_{i}^{\left(S,n\right)}\right)$

 **end for**

${\tilde{h}}_{n}≔mean\left(h_{1}^{n},h_{2}^{n},\ldots ,h_{N}^{n}\right)$

$\sigma \left(n\right)≔std\left(h_{1}^{n},h_{2}^{n},\ldots ,h_{N}^{n}\right)$

**end for**

$\bar{n}≔\underset{n}{\mathrm{arg max}}\frac{{\tilde{h}}_{n-1}}{{\tilde{h}}_{n}}$

$O≔\left\{i\;|\;h_{i}^{\bar{n}}>{\tilde{h}}_{\bar{n}}+c\times \sigma_{\bar{n}}\right\}$

*p*≔|*O*|/*N*

${\bar{n}}^{*}=\bar{n}-\left\lfloor \left(\bar{n}+1\right)\times p\right\rfloor$

(Skip if using original coordinates) Apply a MDS on *D* to create an euclidean space of dimension *d*, resulting $\tilde{X}$

Apply a PCA on $\tilde{X}\backslash O$ to get the main subspace of dimensionality $\bar{n}$

**for** outlier *i* in *O* **do**
 Project *x*_*i*_ on the main subspace, and correct the coordinates of *x*_*i*_ in $\tilde{X}$

**end for**
Recompute the pairwise distance matrix D* from $\tilde{X}$ .
**return** ${\bar{n}}^{*}$ , *O* and D*

The complexity of the algorithm can be briefly evaluated as follows.

1. Given one *n*-simplex, the volume computation has a complexity of $O\left(n^{3}\right)$ . Since we compute the height for *S* simplices and repeat the process for all *E*_dim_ dimensions, the total complexity of this step amounts to $O\left(SNn^{3}E_{\dim}\right)$ ,
2. The complexity of PCA over the regular data points is $O\left(Nd\times \min\left(N,d\right)+d^{3}\right)$ ,
3. The complexity of MDS over the pairwise distance matrix *D* is $O\left(N^{3}\right)$ ,
4. The computation of the corrected distances matrix is $O\left(dN^{2}\right)$ .

Note that with the tested dimensions being smaller than the data dimension (*n*, *E*_dim_ < *d*), and the number of simplices being significantly smaller than the total number of data points (*S* ≪ *N*), the burden of evaluating the simplices (step 1) and correcting outliers (step 4) is in practice less than the cost of the PCA (step 2) and MDS (step 3).

### 2.6 Datasets

#### 2.6.1 Synthetic datasets

**FIGURE 2:**
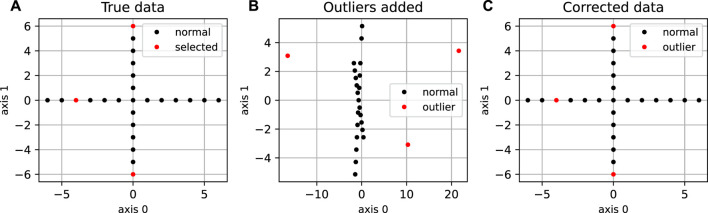
Application of DeCOr-MDS on a cross dataset. **(A)** Original cross dataset. The points selected to be orthogonal outliers are highlighted in red. **(B)** MDS embedding of the original data with an outlying component added to the selected points. **(C)** MDS embedding after preprocessing using DeCOr-MDS. Note that after correction, we recover the original cross structure.

The “cross” dataset (Spence and Lewandowsky, 1989), which is a two-dimensional dataset representing a simple cross structure (Figure 2) was generated with *N* = 25 points, and d* = 2. We introduced orthogonal outliers by randomly sampling three points and by adding a third coordinate of random amplitude to them. Other synthetic datasets were generated by sampling Gaussian-distributed coordinates in the main subspace, and adding some small noise in the whole space with variance between 0.0001 and 0.0003. A fraction *p* of the points was considered to define the orthogonal outliers, with coordinates modified by randomly increasing the coordinate(s) orthogonal to the plane; the amount increased is drawn from a uniform distribution between −30 and 30 or -100 to 100. These datasets were generated for a main subspace of dimension 2, 10 and 40, with *p* = 0.05 and *N* = 200 for dimension d* = 2, *p* = 0.05 and *N* = 1000 for dimension d* = 10, and *p* varying between 0.02 and 0.1 for d* = 40, and *N* = 1, 000. For all the synthetic datasets, the pairwise distance matrix was calculated using the Euclidean distance.

#### 2.6.2 Cell shape dataset

The cell shape dataset contains mouse osteosarcoma 2D imaged cells (Alizadeh et al., 2019), that were processed into a 100 × 2 vector of coordinates that define the cell shape contour, used as a test dataset in the Python package Geomstats (Miolane et al., 2020) (for more details, see also (Miolane et al., 2021) and the associated Github link). We more specifically considered the subset of “DUNN” cells (that denotes a specific lineage) from the control group (no treatment on the cells), which yields 207 cells in total. The pairwise distance matrix of all cell shapes was obtained from the same reference (Miolane et al. (2020; 2021)) using the so-called Square Root Velocity metric that derives from the *L*_2_ distance between velocities of the curves (Srivastava et al., 2010).

#### 2.6.3 HMP dataset

The Human Microbiome Project (HMP) (Turnbaugh et al., 2007) dataset represents the microbiome measured across thousands of human subjects. The human microbiome corresponds to the set of microorganisms associated to the human body, including the gut flora, or the skin microbiota. The data used here corresponds to the HMP1 phase of clinical production. The hypervariable region v13 of ribosomal RNA was sequenced for each sample, which allowed to identify and count each specific microorganism, called phylotype. The processing and classification were performed by the HMP using MOTHUR, and made available as low quality counts (https://www.hmpdacc.org/hmp/HMMCP/) (Turnbaugh et al., 2007). We downloaded this dataset, and subsequently, counts were filtered and normalized as previously described (Legrand, 2017). For our analysis, we also restricted our dataset to samples collected in nose and throat. Samples and phylogenies with less than 10 strictly positive counts were filtered out (Legrand, 2017), resulting in an *n* × *p*-matrix where *n* = 270 samples and *p* = 425 phylotypes. Next, the data distribution was identified with an exponential distribution, by fitting its rate parameter. Normalization was then achieved by replacing the abundances (counts) with the corresponding quantiles. Lastly, the matrix of pairwise distances was obtained using the Euclidean distance.

#### 2.6.4 scRNA-seq dataset

The scRNA-seq dataset contains single-cell transcriptomic profiles from mouse pancreatic cells (raw count data accession number: GSE84133), which were first processed using standard quality control methods from McCarthy et al. (2017). From the gene count matrix, which originally contained 1,886 cells with 13,357 genes, we focused on the cells from Mouse 2, yielding 1,063 cells with 13,357 genes. We further lognormalized the data (Luecken and Theis, 2019) and selected highly variable genes using *scanpy* package (Wolf et al., 2018). This procedure resulted in a normalized gene count matrix of 1,603 cells with 2,601 genes. To obtain a matrix of pairwise distances, we used the Euclidean distance.

## 3 Results

### 3.1 Using n-simplices for orthogonal outlier detection and dimensionality reduction

We propose a robust method to reduce and infer the dimensionality of a dataset from its pairwise distance matrix, by detecting and correcting orthogonal outliers. The method, called *DeCOr-MDS*, can be divided into three sub-procedures detailed in Sections 2.2–2.4, with the overall algorithm provided in Section 2.5. The first procedure detects orthogonal outliers and estimates the subspace dimension using the statistics of simplices that are sampled from the data, using Eqs 4, 5. The second procedure corrects for potential bias in estimated dimension when the fraction of outlier is large. The third procedure corrects the pairwise distance of the original data, by replacing the distance to orthogonal outliers by that to their estimated projection on the main subspace. In the next sections, we report the results obtained upon running the procedure on synthetic and various biological datasets, that demonstrate the performance and accuracy of the method. For all these experiments, we also reported the runtime in Supplementary Table S1, showing how the method can be used in practice with reasonable time on experimental datasets (less than 10 min in our workstation, with x86_64 CPU, 132 GB RAM and 447 GB disk storage).

### 3.2 Performance on synthetic datasets

**FIGURE 3:**
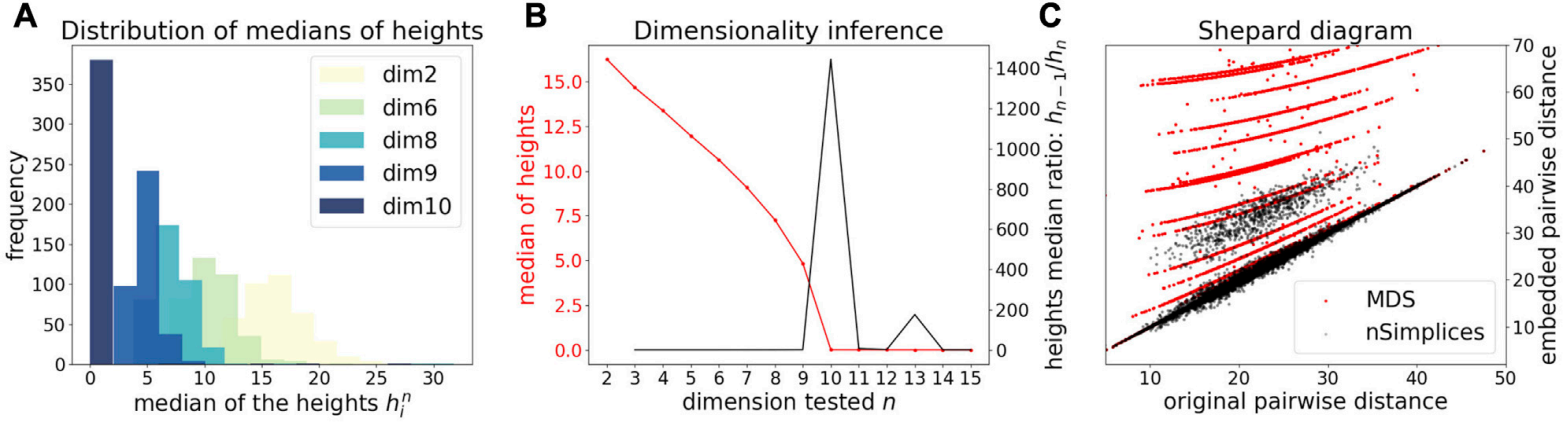
Application of DeCOr-MDS on a synthetic dataset with a main subspace of dimension 10. **(A)** Distribution of median heights per data point *x*_*i*_ as a function of the tested dimension *n*. **(B)** Dimensionality inference based on the ratio of median heights (red curve, see also Eq. 1), with the optimal ratio (black curve) found for the true dimension 10. **(C)** Shepard diagram comparing the pairwise distances between regular points and outliers that are projected to the main subspace (true *δ*_*ij*_), with the same distances obtained after directly running MDS on the original pairwise distance matrix (red dots), or after correcting these distances using our procedure (black dots).

We first illustrate and evaluate the performance of the method on synthetic datasets, (for a detailed description of the datasets and their generation, see the Methods Section 2.6). On a simple dataset of points forming a 2D cross embedded in 3D (Figure 2A), we observed that the MDS is sensitive to the presence of orthogonal outliers and distorts the cross when reducing the data in 2D (Figure 2B). In contrast, our procedure recovers the original geometry of the uncontaminated dataset, with the outliers being correctly projected (Figure 2C). The same results were obtained when sampling regular points from a 2D plane (Supplementary Figure S1). We further tested higher dimensions, and illustrate in Figure 3A how the distribution of heights becomes concentrated around 0, when testing for the true dimension (d* = 10), as suggested in the Methods Section 2.2. As a result, our method allows to infer the main subspace dimension from Eq. 4, as shown in Figure 3B. In addition, the procedure accurately corrects the pairwise distances to orthogonal points with the distances to their projections on the main subspace, as shown in Figure 3C.

**FIGURE 4:**
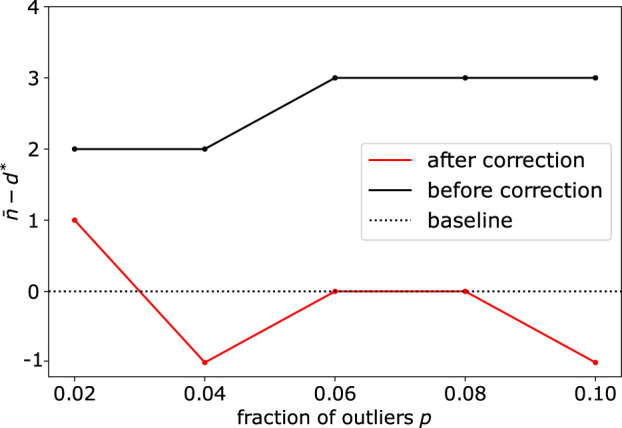
Application of DeCOr-MDS on a dataset with a main subspace of dimension 40: Dimension correction effect versus the fraction of outliers. The vertical axis represents the remaining bias between the inferred and actual dimensions, before and after bias correction. After correction, the differences between the estimated dimensions and the true dimension are always closer to 0 regardless of the fraction of outliers.

When the dimension of the subspace and fraction of outliers get significantly large, we also illustrate the importance of the correction step (see Methods Section 2.3), due to the sampling of simplices that contain several outliers. Upon using synthetic datasets with d* = 40 and varying the number (fraction) of outliers from 20 (2%) to 100 (10%), we observe this bias appearing before correction, with d* being overestimated by 2 or 3 dimensions (Figure 4). Using the debiased estimate n* from Eq. 6 successfully reduced the bias, with an error ≤1 for all the parameters tested.

### 3.3 Application to cell shape data

**FIGURE 5:**
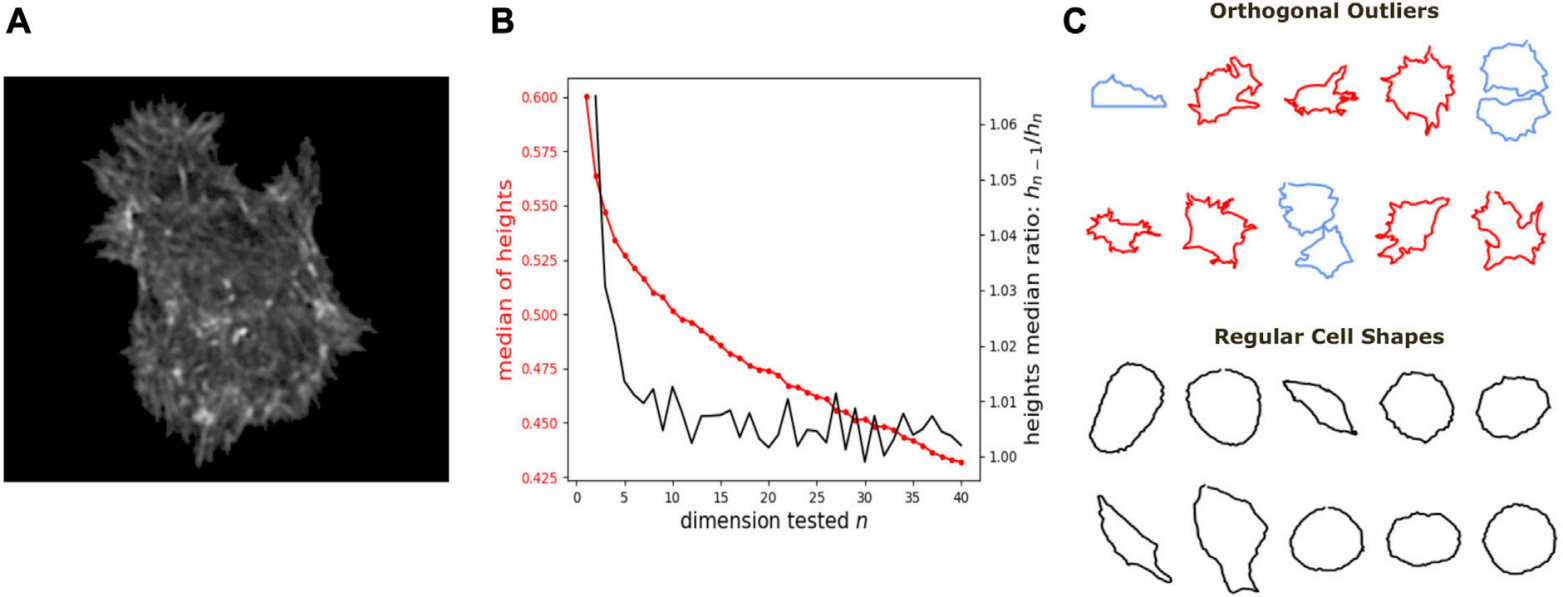
Application of DeCOr-MDS on a cell shapes dataset. **(A)** An example of osteocarcoma cell image obtained from fluorescence microscopy. We process and extract the cell contour in our analysis. **(B)** Dimensionality inference of the dataset obtained from 207 cell shapes using DeCOr-MDS. We estimate the dimension of the main subspace n* = 2. The red line shows the median of heights and the black curve shows the heights median ratios between *h*_*n*−1_ and *h*_*n*_. **(C)** Examples of cell shapes, including regular cells (in black), and orthogonal outliers detected. Among these outliers, we highlight cell shapes that are likely to be invalid due to segmentation errors (in blue), with the other outliers shown in red.

We further show how *DeCOr-MDS* can be broadly applied to biological data, ranging from images to high throughput sequencing. We first studied a dataset of single cell images, from osteosarcoma cells (see Figure 5A), which were processed to extract from their contour a 100 × 2 array of *xy* coordinates representing a discretization of a closed curve (see Dataset Section 2.6). We obtained a pairwise distance matrix on this set of curves by using the so-called *Square Root Velocity* (SRV) metric, which defines a Euclidean distance on the space of velocities that derive from a regular parameterization of the curve (Srivastava et al., 2010; Miolane et al., 2020). Using *DeCOr-MDS*, we found a main subspace of dimension 2 (Figure 5B), with 14 (7%) outliers detected among the 207 cells of this dataset. The comparison between the resulting embedding and that obtained from a simple MDS is shown in Supplementary Figure S2, and reveals that outliers, when uncorrected, affect the embedding coordinates, while our correction mitigates it. By examining in more details the regular and inferred outlier cells (Figure 5C, with all cell shapes shown in Supplementary Figure S3), we found regular observations to approximately describe elliptic shapes, which is in agreement with the dimension found, since ellipses are defined by 2 parameters. One can also visually interpret the orthogonal outliers detected as being more irregular, with the presence of more spikes and small protusions. Interestingly, the procedure also identified as outliers some images containing errors, due to bad cropping or segmentation (with 2 cells shown instead of one), which should thus be removed of the dataset for downstream analysis.

### 3.4 Application to HMP data

As another example of application to biological data, we next considered a dataset from the Human Microbiome Project (HMP). The Human Microbiome Project aims at describing and studying the microbial contribution to the human body. In particular, genes contributed by microbes in the gut are of primary importance in health and disease (Turnbaugh et al., 2007). The resulting data is an array which typically contains the abundance of different elements of the microbiome (typically 10^2^–10^3^), denoted phylotypes, measured in different human subjects. To analyze such high dimensional datasets, dimensionality reduction methods including MDS (often denoted Principal Coordinates Analysis PCoA), are typically applied and used to visualize the data (Brooks et al., 2018; Trevelline and Kohl, 2022; Zhou et al., 2022).

**FIGURE 6:**
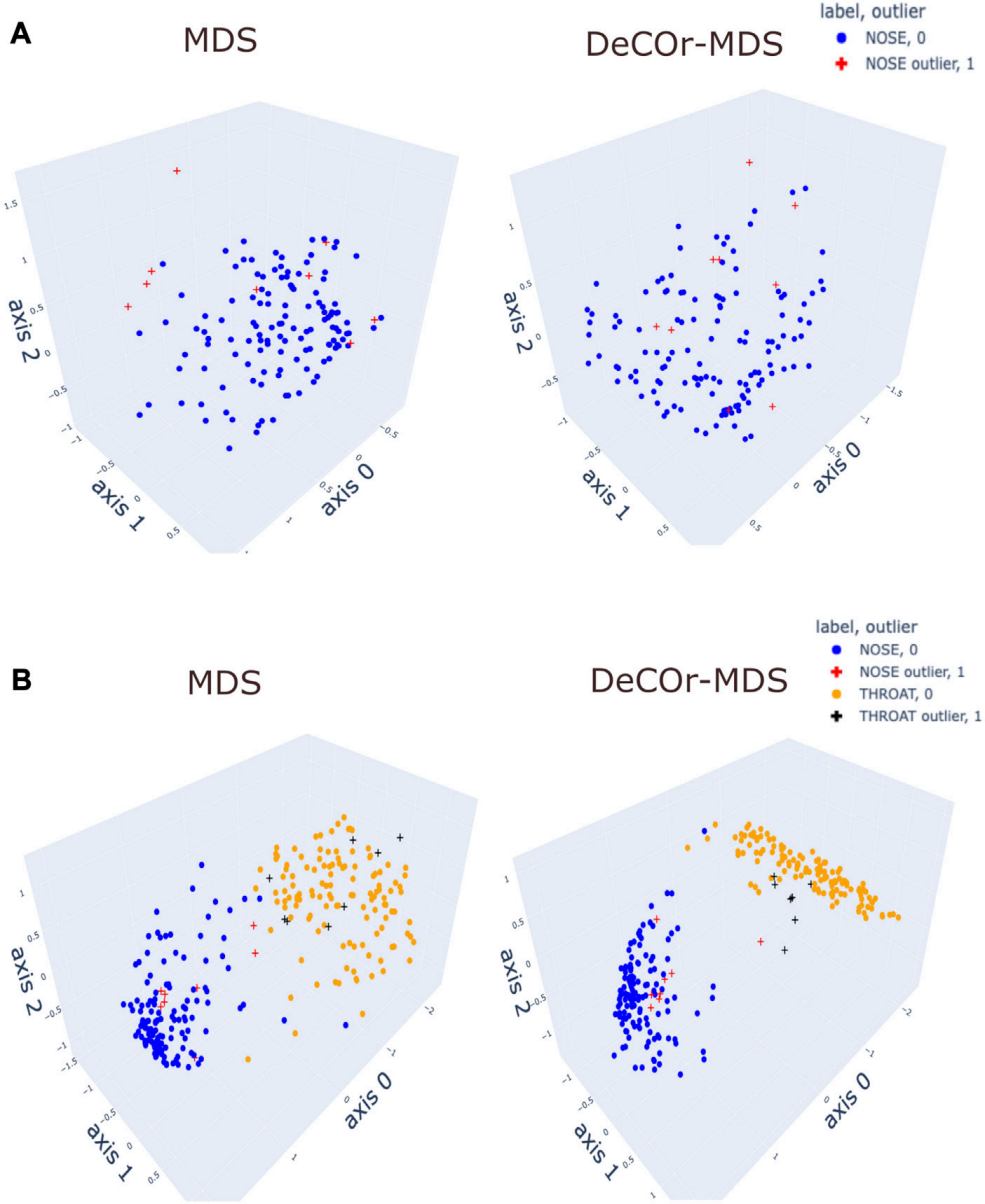
Application of DeCOr-MDS on HMP dataset. **(A)**: Structure restituted on 3 axes using *MDS* (left) and our procedure (right) using data from the nose site. The points marked with cross represent orthogonal outliers detected by *DeCOr-MDS*, which are also put closer to regular points after correction. **(B)** Same comparison as in **(A)** using data from nose and throat. The two clusters formed by nose and throat have a better separation using DeCOr-MDS.

To assess our method incrementally, we restricted first the analysis to a representative specific site (nose), yielding a 136 × 425 array that was further normalized to generate Euclidean pairwise distance matrices (see Material and Methods Section 2.6 for more details). Upon running *DeCOr-MDS*, we estimated the main dimension to be 3, with 9 (6.62%) orthogonal outliers detected, as shown in Figure 6A. This is also supported by another study that the estimated dimension of HMP dataset is 2 or 3 (Tomassi et al., 2021). We also computed the average distance between these orthogonal outliers and the barycenter of regular points in the reduced subspace, and obtained a decrease from 1.21 when using *MDS* to 0.91 when using *DeCOr-MDS*. This decrease suggests that orthogonal outliers get corrected and projected closer to the regular points, to improve the visualization of the data in the reduced subspace, like in our experiments with the synthetic datasets (Figure 2 and Supplementary Figure S2). In Figure 6B, we next aggregated data points from another site (throat) to study how the method performs in this case, yielding a 270 × 425 array that was further normalized to generate Euclidean pairwise distance matrices. As augmenting the dataset brings a separate cluster of data points, the dimension of the main dataset was then estimated to be 2, with 13 (5%) orthogonal outliers detected, as shown in Figure 6B. The average distance between the projected outliers and the barycenter of projected regular points are approximately the same when using *MDS* (1.46) as when using *DeCOr-MDS* (1.45) for nose, and are also approximately the same when using *MDS* (1.75) to when using *DeCOr-MDS* (1.74) for throat. This decrease also suggests that orthogonal outliers get corrected and projected closer to the regular points.

### 3.5 Application to scRNA-seq data

**FIGURE 7:**
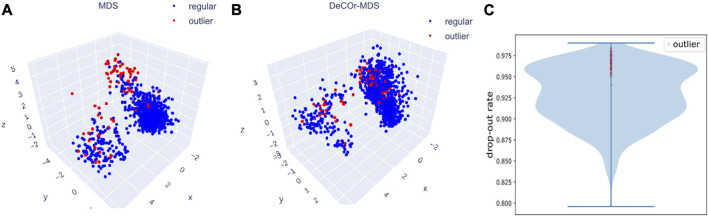
Application of *DeCOr-MDS* on the scRNA-seq dataset. Structure restituted on 3 axes using *MDS* **(A)**, and using *DeCOr-MDS* **(B)**. The red points in **(A)** and **(B)** are outliers detected by *DeCOr-MDS*. The blue points are regular points. **(C)** Violin plot for the drop-out rates for the scRNA-seq dataset, for the top 500 highly expressed genes. Drop-out rates for outliers detected by *DeCOr-MDS* are shown in red.

We further evaluated *DeCOr-MDS* on single cell RNA-seq (scRNA-seq) data. In general, analyzing scRNA seq data requires dimensionality reduction for visualization (including MDS-based methods Canzar et al. (2021); Senabouth et al. (2019)), and specific quality control procedures to mitigate various technical artifacts McCarthy et al. (2017); Luecken and Theis (2019). We applied our method as a potentially relevant tool for this purpose. We applied *DeCOr-MDS* first on a dataset containing the expression level of 1063 cells for 2,601 genes (detailed in Methods Section 2.6). We found the dimension of the main subspace to be 3, with 77 (7%) outliers detected. In Figures 7A,B, we compared the embeddings in 3D using *MDS* and *DeCOr-MDS*. Similarly to the previous experiments, the mean distance between the orthogonal outliers and barycenter of regular points in the reduced subspace decreases when using *DeCOr-MDS* (from 4.22 to 2.51), improving the visualization of regular points. In Figure 7C, we further examined the drop-out rates (indicating zero count for a given gene) of the cells among the top 500highly expressed genes, determined by the median of counts per gene. Among these highly expressed genes, we identified 97.4% of the detected outliers that have drop-out rates greater than 0.95, while this was the case for 27.4% of the regular cells. Upon performing a pairwise *t*-test on the total counts for the top 500 highly expressed genes from the outlier group and the regular cell group, we found that the total counts are significantly different between the two groups (*p*-value < 0.001). Therefore, our method led to detect some outliers associated with high drop-out counts for highly expressed genes, which were not captured by the standard processing and quality control methods used in the first place.

## 4 Discussion

We proposed *DeCOr-MDS*, a novel approach using geometric characteristics to detect dimension, and to correct orthogonal outliers in high dimensional space. That is, to the best of our knowledge, the first statistical tool that addresses the challenge of the presence of orthogonal outliers in high dimensional space. We validated the method using synthetic datasets and demonstrated its potential applications to analyze biological datasets, including cell shape data, count arrays from microbiome data and scRNA-seq data. The visualization and numerical comparison confirmed that *DeCOr-MDS* effectively detects dimensionalities in many instances, corrects orthogonal outliers, and demonstrates superior performance to classical dimension reduction methods.

The notion of simplices is used frequently with the aim of robustness, either to detect the coreness of data [data depth and multivariate median, Liu (1990); Aamari et al. (2021)], or to detect outlying features [detection of extreme directions, Meyer and Wintenberger (2021)]. Simplices can also be used to build a flexible network of points for informative visualization (McInnes et al., 2018). Outlier detection and accommodation have been addressed by a wide array of methods, which can be broadly divided into three categories: 1) robust metrics (Spence and Lewandowsky, 1989; Cayton and Dasgupta, 2006; Oh et al., 2008; Shieh and Hung, 2009; Forero and Giannakis, 2012), 2) robust estimation (Mandanas and Kotropoulos, 2017), or 3) exploiting the characteristics of outliers (Forero and Giannakis, 2012; Blouvshtein and Cohen-Or, 2019). Our method resorts to both (3) by using the geometry of data, and 1) by using the median as centrality estimator. Our method also aims at estimating dimension. A common approach to do so is the screeplot (or elbow) test in principal components analysis, where a notable drop in the proportion of variance (or distance) explained can be taken as a cutoff, and as the most relevant dimension. High-dimensional biological datasets challenge this strategy, because fine-scale structure confounds in practice downstream analyses. Because of this, authors often use an arbitrary large set of 10, or sometimes 20 or 50 components (Astle and Balding, 2009; Barfield et al., 2014; Demmitt et al., 2017; Sakaue et al., 2020; Arciero et al., 2021; Deng et al., 2021). Power analyses based on simulations also provide a way to assess an adequate number of components (Barfield et al., 2014). In this work, we proposed an alternative approach, by exploiting the structure of the dataset to determine essential versus non-essential dimensions.

Limitations of DeCOr-MDS include the non-automated choice of the cutoff parameter *c*. This parameter sets the maximum tolerated number of standard deviations *σ* before a point is considered an outlier. A value for *c* = 3, which corresponds approximately to the 0.1% most extreme points in a Gaussian distribution, may be selected, for instance. Dimension detection is also imperfect for heterogeneous datasets where the distribution of regular points (e.g. with distant clusters may prevent the height criterion for outlier detection to be effective. In this case a possible solution would be to first perform a clustering analysis (for instance k-means) to assess if the distance between clusters is comparable with the distance between the outlier and the main subspace, and if that’s the case separately perform our method on each cluster. There are various potential directions to improve the dimension detection in real datasets of high dimension. This may be achieved by studying the behaviour of the Cayley-Menger determinant, which is central in the procedure, in higher dimensions. One may also associate the height criterion with a distribution criterion (Legrand, 2017), which would be sensitive to clusters or other notable structure, as was apparent in the HMP dataset. Another beneficial improvement would be to reduce computing time, for instance by implementing a parallelized version or using a call to a compiled program. Finally, one could optimize the cutoff parameter *c* automatically, either through a hyperparameter search, or by using a data-driven procedure, during the exploration phase of the algorithm.

## Data Availability

Publicly available datasets were analyzed in this study. This data can be found here: https://osf.io/x5796.
