# Supplementary material for: Orthogonal outlier detection and dimension estimation for improved MDS embedding of biological datasets
Source: Front Bioinform. 2023 Aug 10;3:1211819. doi: 10.3389/fbinf.2023.1211819 (PMC10448701; doi:10.3389/fbinf.2023.1211819)
Supplement: Supplementary file 1 [file DataSheet1.pdf]

Supplementary Material

Wanxin Li[1], Jules Mirone[2,3], Ashok Prasad[4], Nina Miolane[5], Carine Legrand[6,†,*] and Khanh Dao Duc[1,2,†,*]

[1] Department of Computer Science, University of British Columbia, Vancouver, BC V6T 1Z2, Canada
[2] Department of Mathematics, University of British Columbia, Vancouver, BC V6T 1Z4, Canada
[3] Centre de Mathématiques Appliquées, Ecole Polytechnique, route de Saclay, 91128 Palaiseau, France
[4] Department of Chemical and Biological Engineering and School of Biomedical Engineering, Colorado State University, Fort Collins, CO 80523, USA
[5] Department of Electrical and Computer Engineering, University of California, Santa Barbara, CA 93106, USA
[6] Université Paris Cité, Génomes, biologie cellulaire et thérapeutique U944, INSERM, CNRS, F-75010 Paris, France

[†] These authors contributed equally to this work and share last authorship.

[*] Contact and Corresponding Authors: carine.legrand@inserm.fr and kdd@math.ubc.ca

This Supplemental Information contains:

- Supplementary Figures S1-3
- Supplementary Table S1

S1 SUPPLEMENTARY FIGURES

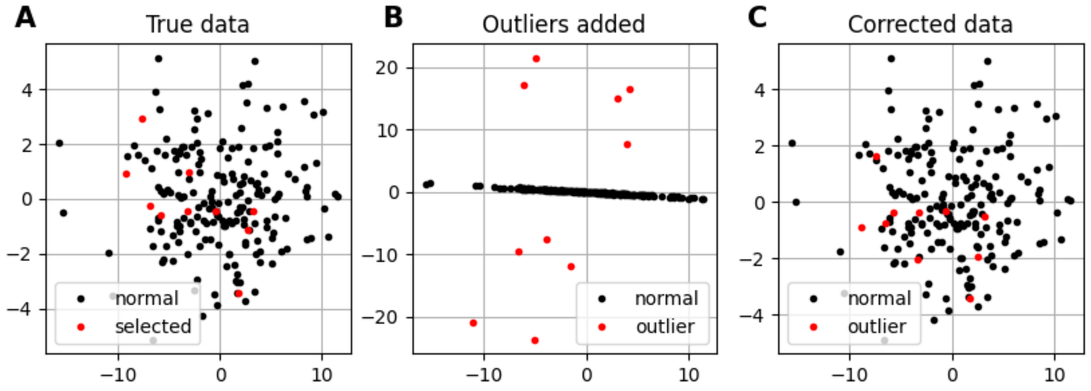

Figure S1: Application of DeCOr-MDS on a synthetic dataset with a main subspace of dimension 2. **A** MDS embedding of the original data; the points selected to be orthogonal outliers are highlighted in red. **B** MDS embedding of the data with an outlying component added to the selected points. **C** MDS embedding of the corrected data using DeCOr-MDS. Note that after correction, we recover the original structure.

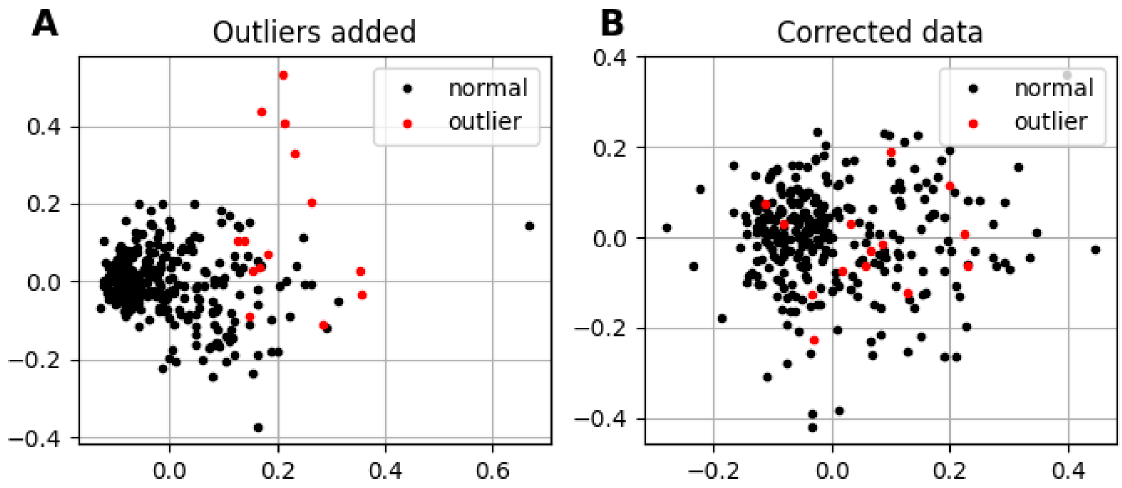

Figure S2: Application of DeCOr-MDS on a cell shape dataset with a main subspace of dimension 2. **A** MDS embedding of the data with an outlying component added to the selected points. **B** MDS embedding of the corrected data using DeCOr-MDS. We notice that the embedding in **A** is distorted, and outlier cells (red points) are corrected to be of the same magnitudes as normal cells.

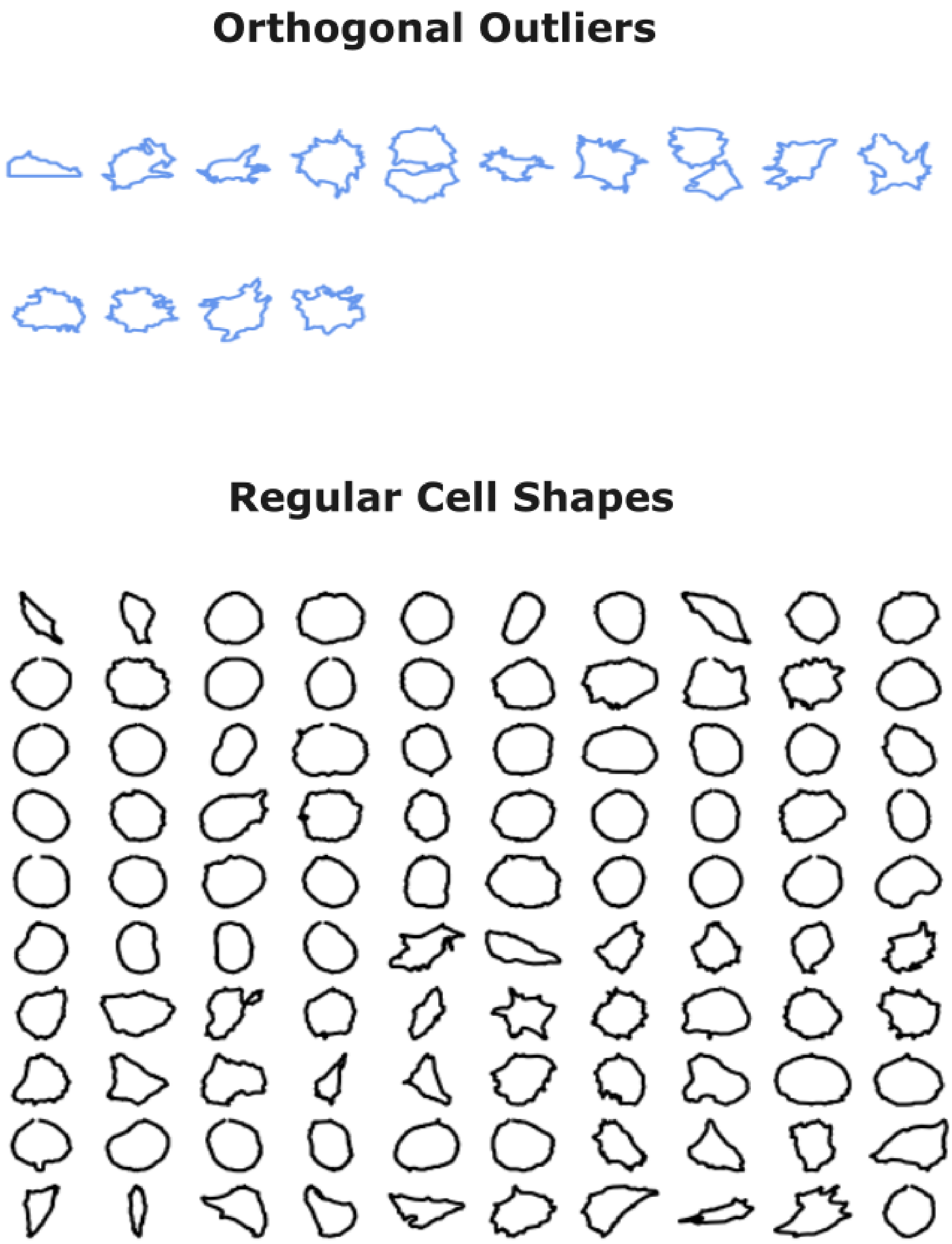

Figure S3: All 14 orthogonal outliers and the first 100 regular cell shapes identified by DeCOr-MDS.

	cross	dimension 2	dimension 10	dimension 40	cell shape	HMP	scRNA-seq
runtime (s)	63.7	71.0	64.9	1417.6	233.5	599.1	160.9

Table S1: Runtime in seconds for the cross dataset, synthetic dataset of dimension 2, cell shape dataset, HMP dataset and scRNA-seq dataset, with $S = 100$ and $c = 3$ (default values). Experiments were perforemed on a workstation with x86_64 CPU, 132 GB RAM and 447 GB disk storage.
